# The association between accelerated biological aging and the risk of osteoarthritis: a cross-sectional study

**DOI:** 10.3389/fpubh.2024.1451737

**Published:** 2024-09-11

**Authors:** Qiang He, Hua Luo, Jie Mei, Zhen Wang, Xin Sun, Ling Wang, Chengxin Xie

**Affiliations:** ^1^Shandong University of Traditional Chinese Medicine, Jinan, China; ^2^Department of Orthopedic, Taizhou Hospital of Zhejiang Province Affiliated to Wenzhou Medical University, Taizhou, China; ^3^QiQiHaEr City Traditional Chinese Medicine Hospital, QiQiHaEr, China; ^4^Nanjing University of Chinese Medicine Affiliated Nanjing Hospital of Traditional Chinese Medicine, Nanjing, China; ^5^The Affiliated Suqian First People’s Hospital of Nanjing Medical University, Suqian, China; ^6^Key Laboratory of Endocrine Glucose & Lipids Metabolism and Brain Aging, Ministry of Education; Department of Endocrinology, Shandong Provincial Hospital Affiliated to Shandong First Medical University, Jinan, China

**Keywords:** epidemiology, biological age, osteoarthritis, NHANES, cross-sectional study

## Abstract

**Background:**

Biological age (BA) offers an effective assessment of true aging state. The progression of Osteoarthritis (OA) is closely associated with an increase in chronological age, the correlation between BA and OA has not been fully elucidated.

**Methods:**

This study analyzed data from the National Health and Nutrition Examination Survey (NHANES) 2005–2018. Thirteen commonly used clinical traits were employed to calculate two measures of BA: the Klemera-Doubal method age (KDM-Age) and phenotypic age (Pheno-Age). The residuals of the regression of these ages based on chronological age were calculated as KDM-Age or Pheno-Age acceleration, respectively. OA was determined through self-reported prior diagnoses. The prevalence of OA across different quartiles of BA was compared using weighted chi-square tests and linear trend tests. The association between BA and OA was assessed using weighted multivariate logistic regression models.

**Results:**

A total of 30,547 participants aged ≥20 years were included in this study, 3,922 (14%) were diagnosed with OA. Participants with OA exhibited higher chronological age, KDM-Age, Pheno-Age, KDM-Age advance, and Pheno-Age advance compared to those without OA (*p < 0.001*). The prevalence of OA significantly increased with higher quartiles of KDM-Age advance and Pheno-Age advance (*P for trend < 0.001*). In the fully adjusted model, compared to the lowest quartile (Q1) of KDM-Age advance, the highest quartile (Q4) was associated with a 36.3% increased risk of OA (OR = 1.363; 95% CI = 1.213 to 1.532, *p < 0.001*). The highest quartile of Pheno-Age advance (Q4) was associated with a 24.3% increased risk of OA compared to Q1 (OR = 1.243; 95% CI = 1.113 to 1.389, *p < 0.001*). In males and young people, no statistical differences were found in OA risk between the highest and the lowest quartiles of KDM-Age advance (*p* = 0.151) and Pheno-Age advance (*p* = 0.057), respectively.

**Conclusion:**

Adults with accelerated biological aging have an increased risk of OA, particularly among females and older adults.

## 1 Introduction

Osteoarthritis (OA) is a common chronic degenerative joint disease characterized by joint dysfunction, pain, and stiffness (1). A study published in the Lancet on global trends and future projections of OA incidence reveals that, as of 2020, OA is the 15th leading cause of disability worldwide, with over 500 million individuals affected globally (2). In the United States, there were significant increasing trends and disparities in self-reported OA prevalence between 2005 and 2018 (3). Currently, the management of early to mid-stage OA primarily involves pharmacological treatments. However, in the terminal stages, joint replacement surgery remains the only effective treatment, though the lifespan of prosthetic joints is limited (4). Consequently, the prevention and control of OA not only require early detection, diagnosis, and treatment but also should focus on reducing controllable risk factors.

The etiology of OA involves multiple factors, including age, genetic susceptibility, obesity, and inflammation, among which age is a primary risk factor (5, 6). The world is gradually entering an era of aging populations, which, as it accelerates, imposes significant economic and healthcare burdens on society (7). Aging is a complex process at the biological level, characterized by the cumulative impact of various molecular and cellular damages over time (8). This leads to a gradual decline in physical and mental capabilities and significantly increases the risk of various types of diseases (9). Addressing health issues related to aging crucially depends on accurately assessing the age of aging and identifying factors that influence aging determinations (10). Research has shown that chronological age (CA) is merely a retrospective age, measuring only the number of years a person has lived (11). Within populations of the same CA, there are significant differences in the rate and apparent degree of aging, making CA a limited and one-sided measure for assessing aging. In contrast, biological aging, which reflects the aging landscape across multiple biological systems (12), is a leading risk factor for most age-related diseases, physical and cognitive impairments, and death (13). Increasing evidence suggests a close relationship between biological aging and geriatric diseases. Unlike CA, biological age (BA) is calculated based on biochemical markers from healthy populations. Using BA can more accurately reflect an individual’s physiological state and the risks associated with aging-related diseases and death. Biological age, as calculated by methods such as the Klemera-Doubal method age (KDM-Age) and phenotypic age (Pheno-Age), relies on a set of readily available clinical measurements and blood test indicators. This provides a comprehensive assessment of a person’s biological age (14). Compared to chronological age, identifying biological age can facilitate timely interventions to prevent disease occurrence, and holds significant clinical importance for the health assessment, management, and prevention of aging-related diseases in the older adults (15).

However, to date, no studies have specifically explored the association between biological age and the risk of OA. This study aims to investigate this association. We hypothesize that patients with OA have higher levels of biological age, and that an increased biological age contributes to the risk of developing OA. This hypothesis builds on the understanding that biological age, which encompasses the aging processes of multiple biological systems, could influence the onset and progression of age-related diseases such as OA. By examining this relationship, we seek to uncover potential biological targets for early intervention and management strategies to mitigate the impact of OA on the aging population.

## 2 Materials and methods

### 2.1 Study design and population

The National Health and Nutrition Examination Survey (NHANES) is a major program of the National Center for Health Statistics (NCHS) in the United States. NHANES is designed as a complex, multistage probability sample to assess the health and nutritional status of adults and children in the United States (16). The data was collected through home interviews and physical examinations. NHANES interviews included information on demographic, socioeconomic, dietary, and health-related parameters. The physical examination includes medical, dental, and physiological measurements; the detailed methodology and protocols have also been described elsewhere1 (3). All adult participants involved in the study signed an informed written consent form. Participants under the age of 18 provided written permission through a parent or guardian. The study received approval from the NCHS Institutional Review Board. NHANES is a publicly available dataset and is not associated with a specific clinical trial; therefore, a Clinical Trial Number is not applicable. Detailed information can be accessed from the website.^1^

**Figure 1:**
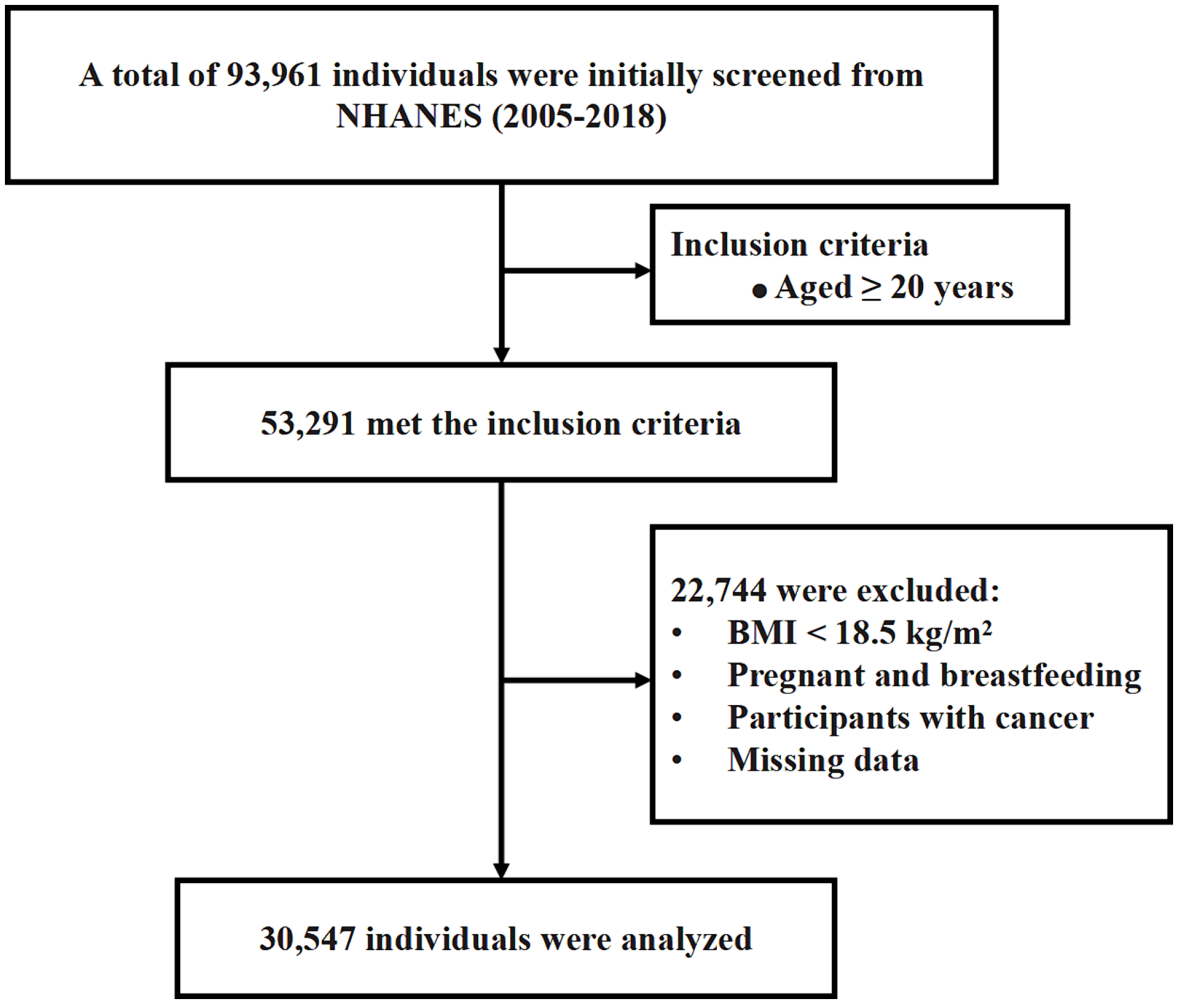
Flow chart of population screening.

This cross-sectional study analyzed data from seven discrete 2-year cycles (2005–2006 through 2017–2018) of the NHANES (3). NHANES only collects osteoarthritis information among adults aged 20 or older. The exclusion criteria were as follows: missing data related to the osteoarthritis health questionnaire; absence of any of the 13 indicators required for calculating biological age; missing demographic data; weight equal to zero; pregnancy or lactation; and participants with cancer. Finally, a total of 30,547 participants were included in the study. The screening process is depicted in Figure 1.

### 2.2 Calculation of biological aging

In this study, biological aging was quantified using two algorithms: KDM-Age and Pheno-Age (14). Specifically, KDM-Age: An individual’s KDM biological age (KDM-BA) prediction corresponds to the age they would be under approximately normal physiological conditions. It is calculated by regressing clinical indicators such as systolic blood pressure, total cholesterol, albumin, glycated hemoglobin, creatinine, C-reactive protein, alkaline phosphatase, and blood urea nitrogen concentrations. Pheno-Age: This is obtained through a multivariate analysis of mortality risk and calculated using clinical indicators including albumin, alkaline phosphatase, creatinine, glucose, C-reactive protein concentrations (CRP), lymphocyte percentage, mean cell volume, red cell distribution width, and white blood cell count. The residuals of the calculated biological age values regressed onto the age at which their biomarkers were measured are defined as KDM-Age acceleration and Pheno-Age acceleration. This is done to evaluate biological aging. A KDM-Age or Pheno-Age acceleration greater than zero is defined as accelerated KDM-Age or accelerated Pheno-Age, respectively, whereas a value less than or equal to zero is considered non-accelerated KDM-Age or Pheno-Age.

### 2.3 Definitions of BMI, WC, and OA

#### 2.3.1 BMI

According to the guidelines set by the World Health Organization in 2008 (17):

1. Underweight: BMI < 18.5 kg/m^2^.
2. Normal range: 18.5 kg/m^2^ ≤ BMI < 25 kg/m^2^.
3. Overweight: 25 kg/m^2^ ≤ BMI < 30 kg/m^2^.
4. Obese: BMI ≥ 30 kg/m^2^.

#### 2.3.2 WC

As per the 26th European Congress on Obesity (ECO) in 2019 (18):

1. For males, a waist circumference ≥ 85 cm is considered above normal.
2. For females, a waist circumference ≥ 80 cm is considered above normal.

#### 2.3.3 OA

The data for the OA variable was indeed collected during the NHANES 2005–2018 cycle. Inclusion of OA and Non-OA participants in this study was based on self-reported OA status from questionnaire surveys (19). The relevant questions included, (1) “Has a doctor or other health professional ever told you that you have arthritis?” Participants who answered “yes” were considered for further classification. (2) “What type of arthritis is this?” Participants who identified “osteoarthritis” met the criteria to be classified as having OA.

### 2.4 Covariates

The covariates included in this study were selected based on their association with OA and their inclusion in similar past research (18). These covariates encompass a range of demographic and health-related variables, Age (Classified according to WHO age categories into three groups: 20–44 years; 45–59 years; and ≥ 60 years), Gender (Categorized as male or female), Race (Divided into Mexican American, Non-Hispanic White, Non-Hispanic Black, Other Hispanic, and Other Race), Education (Grouped as Below High School, High School, and Above High School), Smoking Status (Classified into Never smoker, Former smoker, and Current smoker), Alcohol Consumption (Categorized as <12 drinks/year and ≥ 12 drinks/year), Hypertension (Classified into Hypertension and Non-Hypertension groups), Diabetes [Categorized as DM (diabetes mellitus) and Non-DM]. For detailed measurement techniques for the research variables, the public can access the official website of the Centers for Disease Control and Prevention.^2^ This resource provides comprehensive information on the methodologies and protocols used in the NHANES study, ensuring transparency and accessibility of data collection methods.

### 2.5 Statistical analysis

This study utilized the NHANES database, considering the complexity of NHANES’s sampling design. According to the NHANES Analytical and Reporting Guidelines, the study employed weighted analysis for participant samples from 2005 to 2018 to ensure national representativeness. Data analyses were conducted using R software version 4.2.1 and Empower Stats (version 2.0).

Baseline characteristics were described as follows, Continuous variables were reported as mean ± standard deviation, Categorical variables were presented as frequency (percentage). Differences between the OA and Non-OA groups were evaluated using Student’s t-test for continuous variables and chi-square test for categorical variables. Geometric means and 95% confidence intervals (CIs) for KDM-Age, Pheno-Age, and OA were reported. Survey-weighted linear regression was used for continuous variables, and survey-weighted chi-square tests were utilized for categorical variables to compare baseline characteristics.

The association between KDM-Age, Pheno-Age, and OA risk was analyzed using odds ratios (ORs) and 95% CIs derived from weighted multivariable logistic regression models, fitted with three different levels of adjustment, Model1: A crude model analyzing basic associations, Model 2: Adjusted for age, gender, and ethnicity, Model 3: Further adjusted for education level, smoking status, alcohol consumption, hypertension, and diabetes. To assess dose–response relationships in the three models, KDM-Age and Pheno-Age were modeled both as continuous variables with log transformation and categorically by quartiles (Q1: 25th percentile; Q2: 25th-50th percentile; Q3: 50-75th percentile; Q4: 75th-100th percentile) using restricted cubic splines to explore potential nonlinear associations between KDM-Age, Pheno-Age, and the risk of OA.

All aging indicators were standardized. Subgroup analyses were conducted according to gender. Statistical significance was indicated by two-sided *p*-values less than 0.05.

## 3 Results

### 3.1 Basic characteristics of participants

Among 30,547 participants, the prevalence of OA was 14% (*n* = 3,922). As shown in Table 1, the average age of participants was 45.72 ± 16.69 years, including 14,910 females and 15,637 males. Compared to the Non-OA group, participants in the OA group were older (average age 61.25 ± 12.61 years), predominantly female (64%), Non-Hispanic White (85%), with an education level above high school (66%), smokers (51%), and exhibited higher rates of obesity (BMI ≥30 kg/m^2, WC >0.5 accounting for 80 and 75%, respectively) and hypertension (53%), with statistically significant differences (*p < 0.001*).

**Table 1:** The baseline characteristics of the participants included in the study (*N* = 30,547).

Characteristic	N^1^	Overall *N* = 30,547 (100%)^2^	Non-Osteoarthritis *N* = 26,625 (86%)^2^	Osteoarthritis *N* = 3,922 (14%)^2^	*P*-value^3^
Age (Years)	30,547	45.72 ± 16.69	43.12 ± 15.85	61.25 ± 12.61	<0.001
Age-group (Years)	30,547				<0.001
20–44		14,294 (49%)	13,942 (56%)	352 (9.4%)	
45–59		7,603 (28%)	6,660 (28%)	943 (31%)	
≥60		8,650 (23%)	6,023 (17%)	2,627 (59%)	
Gender	30,547				<0.001
Male		15,637 (51%)	14,194 (53%)	1,443 (36%)	
Female		14,910 (49%)	12,431 (47%)	2,479 (64%)	
Race-group	30,547				<0.001
Non-Hispanic White		13,495 (68%)	10,941 (65%)	2,554 (85%)	
Non-Hispanic Black		5,663 (9.9%)	5,102 (11%)	561 (5.9%)	
Mexican American		5,023 (9.3%)	4,703 (10%)	320 (2.6%)	
Other/multiracial		3,443 (7.2%)	3,212 (7.7%)	231 (4.3%)	
Other Hispanic		2,923 (5.6%)	2,667 (6.1%)	256 (2.2%)	
Education-group	30,547				0.082
Above High School		17,161 (64%)	14,789 (63%)	2,372 (66%)	
Below High School		6,526 (14%)	5,833 (14%)	693 (12%)	
High School		6,860 (22%)	6,003 (22%)	857 (22%)	
Smoke-group	30,547				<0.001
Current smoker		6,113 (19%)	5,443 (20%)	670 (16%)	
Former smoker		7,005 (24%)	5,607 (22%)	1,398 (35%)	
Never smoker		17,429 (57%)	15,575 (58%)	1,854 (49%)	
Drink-group	30,547				<0.001
<12 drinks/year		27,391 (91%)	24,038 (92%)	3,353 (88%)	
≥12 drinks/year		3,156 (8.7%)	2,587 (8.1%)	569 (12%)	
BMI (kg/m^2^)	30,547	28.78 ± 6.48	28.43 ± 6.30	30.81 ± 7.12	<0.001
BMI-group (kg/m^2^)	30,547				<0.001
Normal		9,106 (31%)	8,336 (32%)	770 (20%)	
Obesity		21,441 (69%)	18,289 (68%)	3,152 (80%)	
WC (cm)	30,547	98.63 ± 15.99	97.62 ± 15.74	104.61 ± 16.17	<0.001
WC-group (cm)	30,547				<0.001
Exceeds Standard		16,592 (54%)	13,662 (51%)	2,930 (75%)	
Normal		13,955 (46%)	12,963 (49%)	992 (25%)	
SBP (mmHg)	30,547	121.11 ± 16.36	120.09 ± 15.87	127.20 ± 17.86	<0.001
Total Cholesterol (mg/dL)	30,547	192.00 ± 41.38	190.89 ± 40.13	198.65 ± 47.62	<0.001
Glycated hemoglobin (%)	30,547	5.56 ± 0.89	5.51 ± 0.88	5.81 ± 0.93	<0.001
Albumin (g/dL)	30,547	4.29 ± 0.32	4.30 ± 0.32	4.22 ± 0.31	<0.001
Creatinine (mg/dL)	30,547	2.88 ± 0.24	2.88 ± 0.24	2.89 ± 0.25	0.6
Alkaline phosphatase (U/L)	30,547	66.02 ± 22.02	65.53 ± 21.84	68.92 ± 22.84	<0.001
Blood urea nitrogen (mg/dL)	30,547	13.33 ± 4.94	13.00 ± 4.70	15.31 ± 5.78	<0.001
Serum glucose (mg/dL)	30,547	5.51 ± 1.85	5.44 ± 1.81	5.92 ± 2.05	<0.001
C-reactive protein (mg/dL)	30,547	1.15 ± 0.11	1.15 ± 0.11	1.15 ± 0.12	0.054
Lymphocyte (%)	30,547	30.32 ± 8.00	30.56 ± 7.95	28.85 ± 8.14	<0.001
Mean cell volume (fL)	30,547	89.49 ± 5.33	89.30 ± 5.35	90.61 ± 5.11	<0.001
White blood cell count (1,000 cells/uL)	30,547	7.28 ± 2.22	7.27 ± 2.22	7.37 ± 2.28	0.5
Red cell distribution width (%)	30,547	13.27 ± 1.19	13.23 ± 1.19	13.50 ± 1.13	<0.001
Hypertension-group	30,547				<0.001
Hypertension		9,612 (29%)	7,348 (25%)	2,264 (53%)	
Non-Hypertension		20,935 (71%)	19,277 (75%)	1,658 (47%)	
DM-group	30,547				<0.001
DM		3,299 (8.3%)	2,506 (6.9%)	793 (16%)	
Non-DM		27,248 (92%)	24,119 (93%)	3,129 (84%)	
KDM-Age	30,547	51 ± 19	48 ± 18	69 ± 15	<0.001
KDM-Age advance	30,547	5.2 ± 5.1	4.8 ± 4.9	7.5 ± 5.3	<0.001
Phenoage-Age	30,547	46 ± 18	43 ± 17	62 ± 14	<0.001
Phenoage-Age advance	30,547	0.3 ± 5.2	0.2 ± 5.1	0.9 ± 5.8	<0.001
KDM-Age advance accelerate	30,547	26,349 (87%)	22,708 (86%)	3,641 (94%)	<0.001
Phenoage-Age advance accelerate	30,547	14,662 (46%)	12,559 (45%)	2,103 (50%)	<0.001

1 N not Missing (unweighted).

2 Median (IQR) for continuous; n (%) for categorical.

3 Wilcoxon rank-sum test for complex survey samples; chi-squared test with Rao & Scott’s second-order correction.

Regarding the individual biological age indicators, participants in the OA group had significantly higher values for systolic blood pressure, total cholesterol, glycated hemoglobin, creatinine, alkaline phosphatase, blood urea nitrogen, serum glucose, mean cell volume, and red cell distribution width compared to the Non-OA group (*p < 0.001*). Levels of C-reactive protein and white blood cell count were slightly higher in the OA group than in the Non-OA group, though these differences were not statistically significant. Consistent with the study’s initial hypothesis, participants in the OA group exhibited higher levels of KDM-Age and Pheno-Age, as well as higher values of KDM-Age advance, Pheno-Age advance, KDM-Age acceleration, and Pheno-Age acceleration.

**Figure 2:**
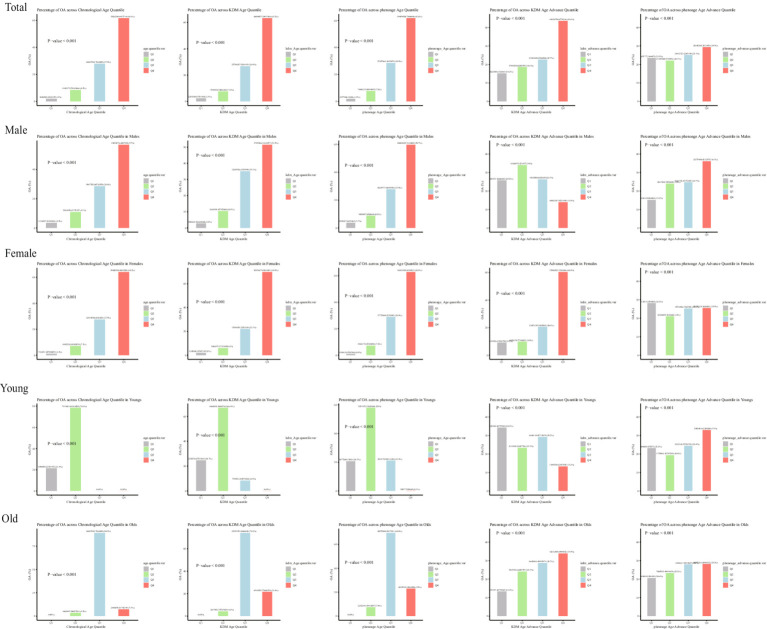
Weighted number of participants with OA.

The baseline characteristics of participants according to quartiles of KDM-Age and Pheno-Age (Supplementary Tables S1, S2, unweighted) revealed significant statistical differences in age, gender, ethnicity, education, smoking, alcohol consumption, BMI, WC, hypertension, and diabetes (*p < 0.001*). Figure 2 shows the weighted number of participants with OA according to quartiles of Chronological-Age, KDM-Age, Pheno-Age, KDM-Age advance, and Pheno-Age advance.

### 3.2 Association of biological aging measures with risk of OA

When participants were categorized into quartiles based on various biological aging indicators, a clear trend of increasing OA prevalence with rising quartiles of KDM-Age and Pheno-Age was observed in the general population (*P for trend < 0.001*), as shown in Supplementary Tables S1, S2.

**Figure 3:**
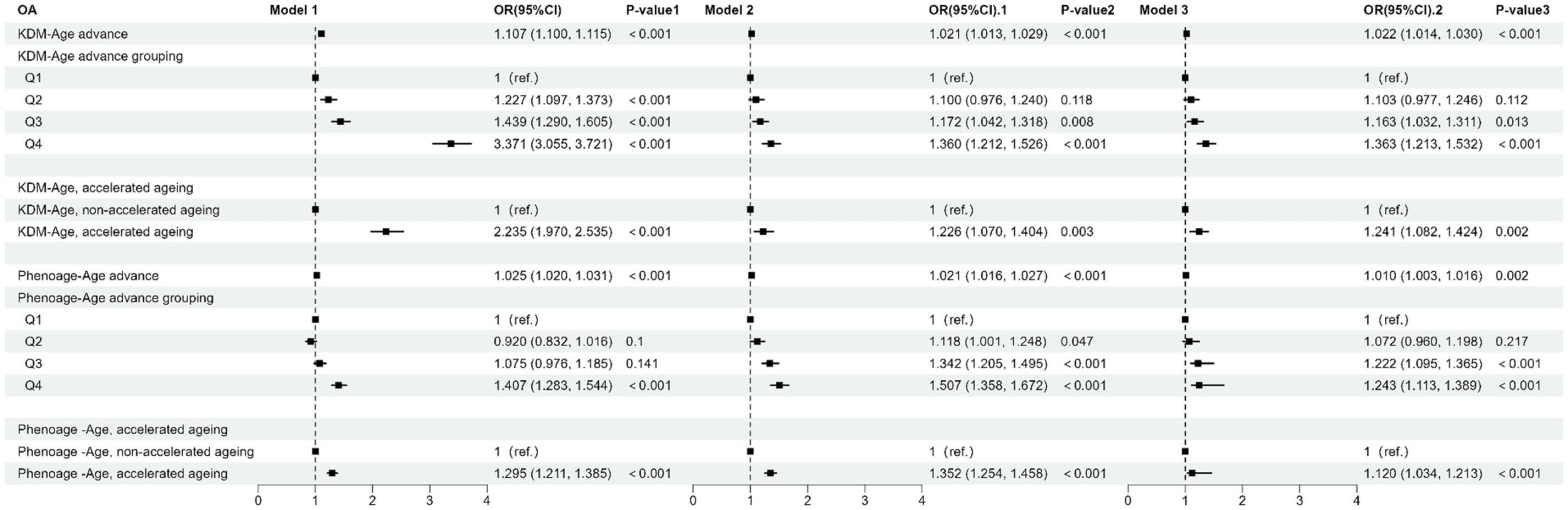
Forest plot of the association between BA and risk of OA.

**Figure 4:**
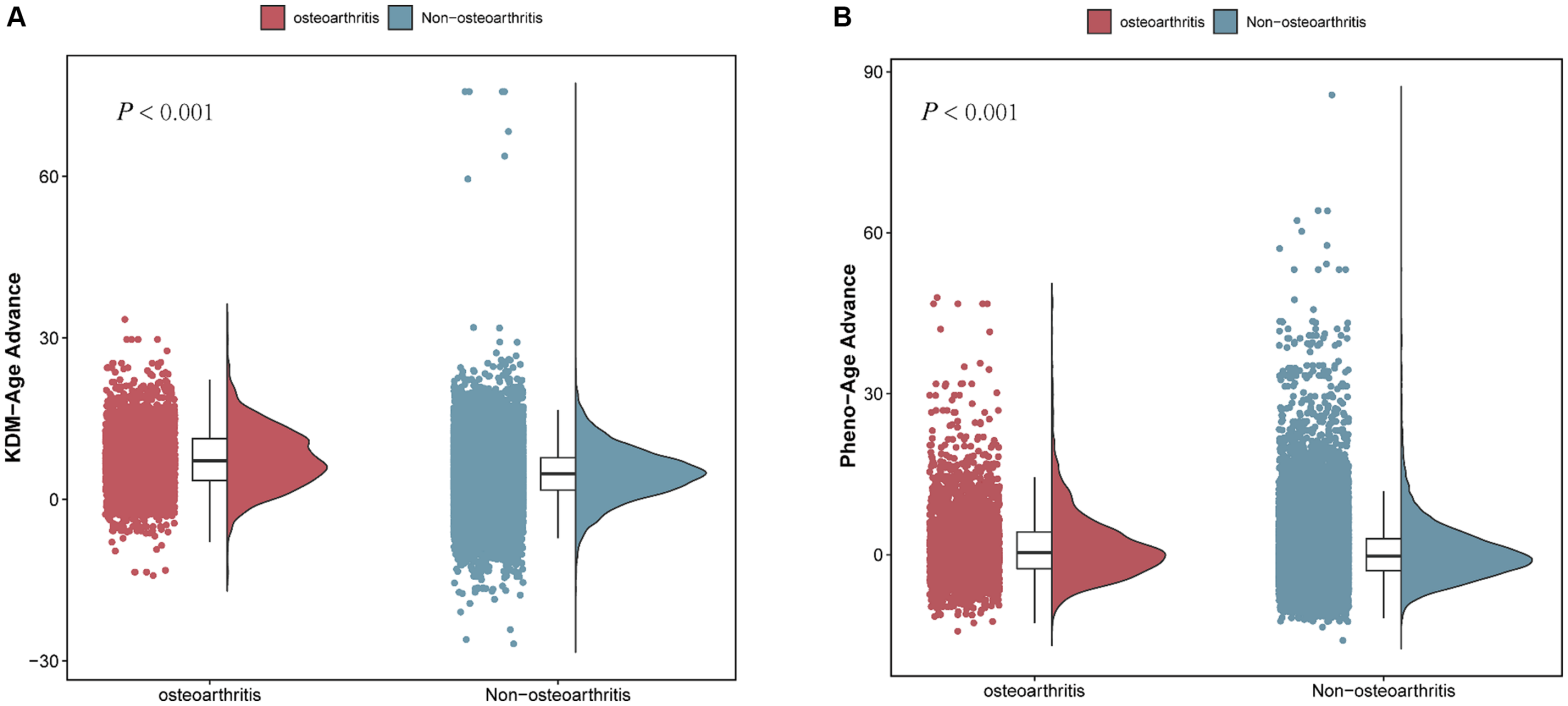
Comparison of BA advance in participants with and without OA. **(A)** KDM-Age advance. **(B)** Pheno-Age advance.

Table 2 and Figure 3 present the results from the weighted multivariable regression analysis, indicating a positive correlation between higher KDM-Age advance and the risk of OA (*p < 0.001*). This association was evident in both the unadjusted Model 1 (OR = 1.107; 95% CI = 1.100–1.115, *p < 0.001*) and Model 2, adjusted for age, gender, and ethnicity (OR = 1.021; 95% CI = 1.013–1.029, *p < 0.001*). In the fully adjusted Model 3, a positive correlation between KDM-Age advance and OA risk persisted (OR = 1.022; 95% CI = 1.014–1.030, *p < 0.001*), suggesting that each unit increase in KDM-Age advance is associated with a 3% increased risk of OA. A sensitivity analysis, transforming KDM-Age advance from a continuous to a categorical variable (quartiles), revealed that compared to the lowest quartile (Q1), the highest quartile (Q4) was associated with a 36.3% higher prevalence of OA in Model 3 (OR = 1.363; 95% CI = 1.213–1.532, *p < 0.001*). A dose–response relationship was also observed across all three models (*P for trend <0.001*), as seen in Figure 4.

**Table 2:** Association of biological aging measures with risk of OA.

	*OR(95% CI) P*-value		
	Model 1	Model 2	Model 3
OA			
KDM-Age advance	1.107 (1.100, 1.115) <0.001	1.021 (1.013, 1.029) <0.001	1.022 (1.014, 1.030) <0.001
KDM-Age advance grouping			
Q1	1(ref.)	1(ref.)	1(ref.)
Q2	1.227 (1.097, 1.373) <0.001	1.100 (0.976, 1.240) 0.118	1.103 (0.977, 1.246) 0.112
Q3	1.439 (1.290, 1.605) <0.001	1.172 (1.042, 1.318) 0.008	1.163 (1.032, 1.311) 0.013
Q4	3.371 (3.055, 3.721) <0.001	1.360 (1.212, 1.526) <0.001	1.363 (1.213, 1.532) <0.001
*P for trend*	<0.001	<0.001	<0.001
KDM-Age, accelerated aging			
KDM-Age, non-accelerated aging	1(ref.)	1(ref.)	1(ref.)
KDM-Age, accelerated aging	2.235 (1.970, 2.535) <0.001	1.226 (1.070, 1.404) 0.003	1.241 (1.082, 1.424) 0.002
*P for trend*	<0.001	<0.001	<0.001
Phenoage-Age advance	1.025 (1.020, 1.031) <0.001	1.021 (1.016, 1.027) <0.001	1.010 (1.003, 1.016) 0.002
Phenoage-Age advance grouping			
Q1	1(ref.)	1(ref.)	1(ref.)
Q2	0.920 (0.832, 1.016) 0.100	1.118 (1.001, 1.248) 0.047	1.072 (0.960, 1.198) 0.217
Q3	1.075 (0.976, 1.185) 0.141	1.342 (1.205, 1.495) < 0.001	1.222 (1.095, 1.365) <0.001
Q4	1.407 (1.283, 1.544) <0.001	1.507 (1.358, 1.672) <0.001	1.243 (1.113, 1.389) <0.001
*P for trend*	<0.001	<0.001	<0.001
Phenoage-Age, accelerated aging			
Phenoage-Age, non-accelerated aging	1(ref.)	1(ref.)	1(ref.)
Phenoage-Age, accelerated aging	1.295 (1.211, 1.385) <0.001	1.352 (1.254, 1.458) <0.001	1.120 (1.034, 1.213) <0.001
*P for trend*	<0.001	<0.001	<0.001

Table 2 and Figure 4B show that a higher Pheno-Age advance also correlates positively with OA risk (*p* < 0.001). This relationship holds in the unadjusted Model 1 (OR = 1.025; 95% CI = 1.020–1.031, *p < 0.001*) and Model 2 (OR = 1.021; 95% CI = 1.016–1.027, *p < 0.001*). In Model 3, a positive correlation between Pheno-Age advance and OA risk continues (OR = 1.010; 95% CI = 1.003–1.016, *p < 0.001*), indicating that each unit increase in Pheno-Age advance is associated with a 0.3% higher prevalence of OA. Further sensitivity analysis with Pheno-Age advance as a categorical variable revealed that compared to Q1, the highest quartile (Q4) has 24.3% higher prevalence of OA in Model 3 (OR = 1.243; 95% CI = 1.113–1.389, *p < 0.001*). A dose–response relationship was also observed in all three models (*P for trend < 0.001*), as shown in Figure 4.

Additionally, both accelerated KDM-Age and accelerated Pheno-Age showed a positive correlation with OA risk (*p < 0.001*). In the fully adjusted Model 3, accelerated KDM-Age was associated with a 24.1% higher prevalence of OA (OR = 1.241; 95% CI = 1.082–1.424, *p < 0.001*), and accelerated Pheno-Age was associated with a 12% higher prevalence (OR = 1.120; 95% CI = 1.034–1.213, *p < 0.001*). A dose–response relationship was evident across the three models (*P for trend <0.001*), as depicted in Figure 4.

### 3.3 Relationship between biological aging and OA risk across different genders

Figure 2 presents the number of individuals with OA across different age and sex groups, categorized by quartiles of Chronological-Age, KDM-Age, Pheno-Age, KDM-Age advance, and Pheno-Age advance. The results indicate that both men and women show similar trends in OA prevalence across Chronological Age, KDM-Age, Pheno-Age, and KDM-Age advance. In females, a higher prevalence of OA was found in the highest quartile (Q4) compared to the lowest quartile (Q1; all *p < 0.001*). However, this trend was disappeared in Pheno-Age advance group. Older adults in the highest quartile (Q4) of Chronological Age, KDM-Age, Pheno-Age, and KDM-Age advance have significantly higher OA prevalence compared to those in the lowest quartile (Q1; all *p < 0.001*). For Pheno-Age advance, the trend is reversed.

Table 3 presents the inconsistent relationships between KDM-Age advance, Pheno-Age advance, and OA risk across different genders. The results from the fully adjusted Model 3 show that compared to the lowest quartile (Q1), male and young participants in the highest quartile (Q4) of KDM-Age advance and Pheno-Age advance did not exhibit a statistically significant higher prevalence of OA (male: *p = 0.151 and p = 0.057*, respectively; young: *p = 0.214 and p = 0.091*, respectively), nor was there a dose–response relationship (*P for trend > 0.05*). In contrast, the results for female participants were quite different.

**Table 3:** Subgroup analysis for the association between biological aging and OA.

	*OR(95% CI) P-*value		
	Model 1	Model 2	Model 3
OA			
Male			
KDM-Age advance	0.943 (0.929, 0.957) <0.001	1.020 (1.004, 1.035) 0.125	1.009 (0.994, 1.025) 0.248
KDM-Age advance grouping			
Q1	1(ref.)	1(ref.)	1(ref.)
Q2	0.719 (0.623, 0.830) <0.001	1.077 (0.925, 1.254) 0.337	1.073 (0.919, 1.252) 0.371
Q3	0.529 (0.455, 0.615) <0.001	1.007 (0.856, 1.184) 0.937	0.967 (0.820, 1.141) 0.693
Q4	0.542 (0.456, 0.646) <0.001	1.316 (1.089, 1.592) 0.004	1.154 (0.949, 1.404) 0.151
*P for trend*	<0.001	0.041	0.469
Female			
KDM-Age advance	1.152 (1.143, 1.161) <0.001	1.012 (1.002, 1.022) 0.018	1.017 (1.007, 1.027) 0.001
KDM-Age advance grouping			
Q1	1(ref.)	1(ref.)	1(ref.)
Q2	2.028 (1.687, 2.438) <0.001	1.024 (0.835, 1.254) 0.822	1.030 (0.839, 1.266) 0.775
Q3	4.316 (3.658, 5.092) <0.001	1.203 (0.991, 1.460) 0.061	1.252 (1.029, 1.523) 0.024
Q4	9.241 (7.984, 10.696) <0.001	1.226 (1.016, 1.479) 0.033	1.319 (1.090, 1.596) 0.004
*P for trend*	<0.001	<0.001	<0.001
Male			
Phenoage-Age advance	1.046 (1.037, 1.054) <0.001	1.021 (1.012, 1.030) <0.001	1.015 (1.005, 1.025) 0.002
Phenoage-Age advance grouping			
Q1	1(ref.)	1(ref.)	1(ref.)
Q2	1.158 (0.964, 1.391) 0.116	1.060 (0.875, 1.284) 0.055	1.049 (0.864, 1.273) 0.629
Q3	1.195 (0.999, 1.429) 0.051	1.064 (0.882, 1.283) 0.051	1.024 (0.847, 1.239) 0.807
Q4	1.903 (1.606, 2.254) <0.001	1.335 (1.116, 1.596) 0.001	1.200 (0.994, 1.450) 0.057
*P for trend*	<0.001	0.002	0.132
Female			
Phenoage-Age advance	1.022 (1.015, 1.029) <0.001	1.025 (1.017, 1.032) <0.001	1.009 (1.001, 1.017) 0.031
Phenoage-Age advance grouping			
Q1	1(ref.)	1(ref.)	1(ref.)
Q2	0.965 (0.854, 1.091) 0.571	1.110 (0.968, 1.272) 0.135	1.059 (0.923, 1.216) 0.412
Q3	1.334 (1.184, 1.504) <0.001	1.553 (1.356, 1.777) <0.001	1.383 (1.204, 1.588) <0.001
Q4	1.471 (1.309, 1.653) <0.001	1.629 (1.427, 1.858) <0.001	1.283 (1.113, 1.478) <0.001
*P for trend*	<0.001	<0.001	<0.001

In the fully adjusted Model 3 for females, compared to Q1, the prevalence of OA was significantly higher for those in the highest quartile (Q4) of KDM-Age advance and Pheno-Age advance, with increases of 31.9% (OR = 1.319; 95%CI = 1.090–1.596, *p = 0.004*) and 28.3% (OR = 1.283; 95%CI = 1.113–1.478, *p < 0.001*), respectively. Additionally, a dose–response relationship was observed in all three models (*P for trend < 0.001*), indicating that as the level of biological aging increased, so did the risk of OA, particularly among female participants. This suggests that biological age may have a more pronounced impact on OA risk in females than in males, emphasizing the need for gender-specific approaches in managing and preventing OA.

## 4 Discussion

This study incorporated data from 30,547 participants from the NHANES database spanning 2005–2018, including 14,910 females and 15,637 males, with 3,922 (14%) reporting OA. It focused on exploring the relationships between two measures of biological age, KDM-Age and Pheno-Age, and their adjusted counterparts, KDM-Age advance and Pheno-Age advance, with the risk of OA. The findings indicated that, compared to the non-OA participants, those with OA had higher KDM-Age, Pheno-Age, KDM-Age advance, and Pheno-Age advance. After accounting for confounding factors that influence the risk of OA, it was evident that an increase in KDM-Age advance and Pheno-Age advance was associated with a higher risk of OA, particularly among participants aged ≥60 years and females. This study contributes significantly to our understanding of how biological aging correlates with the development and severity of OA.

To date, this study is the first to utilize the NHANES database to analyze the correlation between biological age and the risk of OA. OA is a degenerative joint disease with particularly high prevalence and disability rates among populations over the age of 60, reaching up to 50 and 53%, respectively (20). Our findings corroborate this, showing an OA incidence of 59% (2,627/3922) in participants aged over 60. The primary pathological mechanisms of OA include cartilage degradation, bone proliferation, osteophyte formation, joint space narrowing, and degenerative inflammation (21). It is currently believed that factors such as age, gender, obesity, and inflammation are closely linked to OA (22). Our research confirms these perspectives, with women representing 64% (2,479/3922) of OA cases, significantly higher than men, and obese participants constituting 80% (3,152/3922), markedly higher than their non-obese counterparts. While age is recognized as the primary risk factor for the onset and progression of OA, and its development is clearly linked to an increase in chronological age, individuals of the same chronological age can still present divergently as either affected or not by OA. To further explore what drives these differences, we have introduced the indicator of BA.

BA integrates multiple aging-related biomarkers, reflecting the overall aging of the body and can prospectively predict age-related diseases (23). Factors such as genetics, medications, and lifestyle can influence BA, offering a unique perspective for intervening in age-related diseases (24). Recent years have seen significant advancements in the assessment of BA, with various physiological system indicators—such as renal, cardiovascular, and skeletal—showing different biological aging trajectories (25). However, previous studies assessing biological age in individuals over 60 have been limited and shown lower predictive accuracy, often involving comparatively younger populations. Some aging markers only manifest their effects distinctly in older adults. For example, research by Freund and colleagues has shown that serum IL-6 levels are typically low and difficult to detect in younger populations, but in those over 60, stimulated peripheral lymphocytes produce higher levels of IL-6 (26). Similarly, Cesari and others have found in a study of 1,061 older individuals from the Chianti region of Italy, that high serum levels of IL-6 and CRP are associated with poorer physical function in the older adults (27). In this study, 13 clinical indicators were used to comprehensively assess BA. The results demonstrated a positive correlation between higher KDM-Age advance and Pheno-Age advance with the risk of OA (*p < 0.001*). Furthermore, a dose–response relationship was observed across all three models (*P for trend < 0.001*), underscoring the importance of BA as a predictor of OA risk.

Interestingly, our subgroup analysis by gender revealed differing trends between male and female participants in Model 3, fully adjusted. Compared to the lowest quartile (Q1), male participants in the highest quartile (Q4) for KDM-Age advance and Pheno-Age advance showed no statistically significant difference in the risk of developing OA (*p = 0.151, p = 0.057*), nor was there a dose–response relationship (*P for trend >0.05*). In contrast, the results for female participants were significantly different. Epidemiological studies have found that the prevalence of OA in women (4.8%) is approximately twice that of men (2.8%), with onset occurring at an earlier age in women (28). This outcome suggests that women may be more susceptible to the effects of estrogen levels. Research by Bronikowski et al. (29) supports the notion that gender differences in aging occur in many animal species, including differences in lifespan, the onset and progression of age-related declines, and the physiological and molecular markers of aging. These findings highlight the complexity of gender-specific aging processes and their implications for diseases such as OA.

The findings from our study align with the results reported by Xu and Wu (3), who identified significant increasing trends and disparities in OA prevalence among US adults between 2005 and 2018. This trend is particularly pronounced among women, a finding that is consistent with our observation that females and older adults exhibit higher levels of biological age acceleration, which correlates with an increased risk of OA. The consistent rise in OA prevalence can be partly attributed to increasing obesity rates, a major risk factor for OA, as highlighted in both studies. These insights underscore the importance of addressing modifiable risk factors such as obesity and implementing targeted interventions to manage biological aging processes, particularly in high-risk groups like women and the older adults.

Our study is the first to explore the relationship between biological age and OA in a large sample size based on the NHANES database. At the same time, the findings were strengthened by applying survey weights to ensure the generalizability of our findings to US adults through a nationally representative sample, as well as by integrating NHANES data to obtain comprehensive data on a large number of important covariates. Moreover, age was adjusted for when calculating biological age acceleration, while adjusting for age as a confounding factor, and relatively detailed analyses of subgroups were conducted. However, there are some limitations to our study. First, as a cross-sectional analysis, the ability to establish clear causal relationships is inherently limited. Second, some of the variables were obtained through questionnaires and self-reports, which are prone to bias. Third, we did not analyze arthritis body measures from the NHANES 2009–2010 cycle, missing a potential source for validity checks. Finally, due to the limitations of the study population, we did not fully consider the relationship between biological age and OA among different ethnic groups. In the future, cross-sectional studies involving different ethnic subgroups are crucial to provide stronger evidence for our findings.

## 5 Conclusion

This study provides new insights into the relationship between biological age and the risk of OA, adults with accelerated biological aging are associated with higher prevalence of OA, especially females and the older adults. With the trend of population aging, focusing on the prevention and treatment of OA is a public health priority. Interventions to slow biological aging in older adults and females are important to reduce the burden of disease.

## Data Availability

Publicly available datasets were analyzed in this study. This data can be found here: https://wwwn.cdc.gov/nchs/nhanes/Default.aspx.

## Footnotes

^1^ https://www.cdc.gov/nchs/nhanes/

^2^ http://www.cdc.gov/nchs/nhanes
