# Supplementary material for: The association between accelerated biological aging and the risk of osteoarthritis: a cross-sectional study
Source: Front Public Health. 2024 Sep 11;12:1451737. doi: 10.3389/fpubh.2024.1451737 (PMC11423293; doi:10.3389/fpubh.2024.1451737)
Supplement: Supplementary file 2 [file Table_1.DOCX]

Table S1 KDM-Age advance quantile

| Characteristic | N^1^ | Overall  N = 30547 (100%)^2^ | Q1  N = 8005 (25%)^2^ | Q2  N = 7419 (25%)^2^ | Q3  N = 7425 (25%)^2^ | Q4  N = 7698 (25%)^2^ | P Value^3^ |
| --- | --- | --- | --- | --- | --- | --- | --- |
| Age (Years) | 30,547 | 45.72 ± 16.69 | 40.82 ± 16.54 | 44.30 ± 15.14 | 44.58 ± 14.78 | 53.17 ± 17.60 | <0.001 |
| Age-group (Years) | 30,547 |  |  |  |  |  | <0.001 |
| 20-44 |  | 14,294 (49%) | 4,841 (64%) | 3,623 (51%) | 3,563 (50%) | 2,267 (31%) |  |
| 45-59 |  | 7,603 (28%) | 1,400 (20%) | 2,155 (32%) | 2,225 (33%) | 1,823 (28%) |  |
| ≥60 |  | 8,650 (23%) | 1,764 (17%) | 1,641 (17%) | 1,637 (17%) | 3,608 (41%) |  |
| Gender | 30,547 |  |  |  |  |  | <0.001 |
| Male |  | 15,637 (51%) | 3,088 (36%) | 4,782 (65%) | 4,817 (65%) | 2,950 (38%) |  |
| Female |  | 14,910 (49%) | 4,917 (64%) | 2,637 (35%) | 2,608 (35%) | 4,748 (62%) |  |
| Race-group | 30,547 |  |  |  |  |  | <0.001 |
| Non-Hispanic White |  | 13,495 (68%) | 3,096 (63%) | 3,213 (68%) | 3,379 (69%) | 3,807 (73%) |  |
| Non-Hispanic Black |  | 5,663 (9.9%) | 1,891 (14%) | 1,286 (8.7%) | 1,250 (8.6%) | 1,236 (8.2%) |  |
| Mexican American |  | 5,023 (9.3%) | 1,315 (9.7%) | 1,338 (10%) | 1,195 (9.3%) | 1,175 (8.1%) |  |
| Other/multiracial |  | 3,443 (7.2%) | 948 (7.8%) | 932 (8.0%) | 827 (6.8%) | 736 (6.2%) |  |
| Other Hispanic |  | 2,923 (5.6%) | 755 (5.9%) | 650 (5.4%) | 774 (6.1%) | 744 (4.9%) |  |
| Education-group | 30,547 |  |  |  |  |  | <0.001 |
| Above High School |  | 17,161 (64%) | 4,781 (67%) | 4,330 (67%) | 4,102 (62%) | 3,948 (59%) |  |
| Below High School |  | 6,526 (14%) | 1,588 (13%) | 1,558 (13%) | 1,615 (15%) | 1,765 (15%) |  |
| High School |  | 6,860 (22%) | 1,636 (20%) | 1,531 (20%) | 1,708 (23%) | 1,985 (26%) |  |
| Smoke-group | 30,547 |  |  |  |  |  | <0.001 |
| Current smoker |  | 6,113 (19%) | 1,323 (16%) | 1,397 (18%) | 1,771 (22%) | 1,622 (21%) |  |
| Former smoker |  | 7,005 (24%) | 1,709 (21%) | 1,786 (24%) | 1,718 (24%) | 1,792 (26%) |  |
| Never smoker |  | 17,429 (57%) | 4,973 (63%) | 4,236 (58%) | 3,936 (54%) | 4,284 (53%) |  |
| Drink-group | 30,547 |  |  |  |  |  | <0.001 |
| <12 drinks/year |  | 27,391 (91%) | 7,317 (93%) | 6,631 (91%) | 6,576 (90%) | 6,867 (90%) |  |
| ≥12 drinks/year |  | 3,156 (8.7%) | 688 (6.7%) | 788 (8.9%) | 849 (9.8%) | 831 (9.7%) |  |
| BMI (kg/m^2^) | 30,547 | 28.78 ± 6.48 | 29.04 ± 7.14 | 28.12 ± 5.75 | 28.82 ± 6.17 | 29.11 ± 6.72 | 0.004 |
| BMI-group(kg/m^2^) | 30,547 |  |  |  |  |  | 0.5 |
| Normal |  | 9,106 (31%) | 2,441 (32%) | 2,245 (31%) | 2,183 (30%) | 2,237 (30%) |  |
| Obesiy |  | 21,441 (69%) | 5,564 (68%) | 5,174 (69%) | 5,242 (70%) | 5,461 (70%) |  |
| WC (cm) | 30,547 | 98.63 ± 15.99 | 97.77 ± 16.69 | 97.71 ± 14.49 | 99.37 ± 15.86 | 99.65 ± 16.72 | 0.002 |
| WC-group (cm) | 30,547 |  |  |  |  |  | <0.001 |
| Exceeds Standard |  | 16,592 (54%) | 4,317 (54%) | 3,581 (48%) | 3,790 (51%) | 4,904 (63%) |  |
| Normal |  | 13,955 (46%) | 3,688 (46%) | 3,838 (52%) | 3,635 (49%) | 2,794 (37%) |  |
| SBP (mmHg) | 30,547 | 121.11 ± 16.36 | 117.85 ± 15.90 | 120.24 ± 15.44 | 120.48 ± 14.76 | 125.89 ± 18.09 | <0.001 |
| Total Cholesterol (mg/dL) | 30,547 | 192.00 ± 41.38 | 185.41 ± 37.86 | 191.83 ± 39.17 | 191.64 ± 41.10 | 199.13 ± 45.79 | <0.001 |
| Glycated hemoglobin (%) | 30,547 | 5.56 ± 0.89 | 5.48 ± 0.85 | 5.49 ± 0.81 | 5.54 ± 0.89 | 5.72 ± 0.98 | <0.001 |
| Albumin (g/dL) | 30,547 | 4.29 ± 0.32 | 4.22 ± 0.41 | 4.38 ± 0.27 | 4.30 ± 0.25 | 4.26 ± 0.30 | <0.001 |
| Creatinine (mg/dL) | 30,547 | 2.88 ± 0.24 | 2.84 ± 0.24 | 2.91 ± 0.23 | 2.91 ± 0.22 | 2.88 ± 0.25 | <0.001 |
| Alkaline phosphatase (U/L) | 30,547 | 66.02 ± 22.02 | 63.27 ± 24.25 | 64.42 ± 18.80 | 65.98 ± 21.31 | 70.40 ± 22.70 | <0.001 |
| Blood urea nitrogen (mg/dL) | 30,547 | 13.33 ± 4.94 | 12.29 ± 4.70 | 13.33 ± 4.62 | 13.44 ± 4.75 | 14.25 ± 5.44 | <0.001 |
| Serum glucose (mg/dL) | 30,547 | 5.51 ± 1.85 | 5.37 ± 1.71 | 5.40 ± 1.63 | 5.51 ± 1.85 | 5.76 ± 2.16 | <0.001 |
| C-reactive protein (mg/dL) | 30,547 | 1.15 ± 0.11 | 1.17 ± 0.14 | 1.13 ± 0.07 | 1.14 ± 0.07 | 1.16 ± 0.13 | <0.001 |
| Lymphocyte (%) | 30,547 | 30.32 ± 8.00 | 32.03 ± 8.20 | 31.07 ± 7.44 | 29.29 ± 7.48 | 28.87 ± 8.43 | <0.001 |
| Mean cell volume (fL) | 30,547 | 89.49 ± 5.33 | 88.63 ± 5.93 | 89.60 ± 5.15 | 89.70 ± 5.00 | 90.02 ± 5.11 | <0.001 |
| White blood cell count  (1000 cells/uL) | 30,547 | 7.28 ± 2.22 | 6.93 ± 1.96 | 6.97 ± 1.87 | 7.32 ± 2.04 | 7.91 ± 2.77 | <0.001 |
| Red cell distribution width (%) | 30,547 | 13.27 ± 1.19 | 13.43 ± 1.43 | 13.18 ± 1.16 | 13.17 ± 1.03 | 13.29 ± 1.06 | <0.001 |
| Hypertension-group | 30,547 |  |  |  |  |  | <0.001 |
| Hypertension |  | 9,612 (29%) | 2,110 (23%) | 2,074 (27%) | 2,123 (27%) | 3,305 (39%) |  |
| Non-Hypertension |  | 20,935 (71%) | 5,895 (77%) | 5,345 (73%) | 5,302 (73%) | 4,393 (61%) |  |
| DM-group | 30,547 |  |  |  |  |  | <0.001 |
| DM |  | 3,299 (8.3%) | 728 (6.9%) | 693 (7.2%) | 746 (8.2%) | 1,132 (11%) |  |
| Non-DM |  | 27,248 (92%) | 7,277 (93%) | 6,726 (93%) | 6,679 (92%) | 6,566 (89%) |  |
| KDM-Age | 30,547 | 51 ± 19 | 40 ± 18 | 48 ± 15 | 51 ± 15 | 65 ± 19 | <0.001 |
| Phenoage-Age | 30,547 | 46 ± 18 | 41 ± 18 | 44 ± 17 | 45 ± 16 | 54 ± 18 | <0.001 |
| Phenoage-Age advance | 30,547 | 0.3 ± 5.2 | 0.1 ± 5.3 | -0.4 ± 4.6 | 0.5 ± 4.7 | 0.9 ± 6.2 | <0.001 |
| KDM-Age advance accelerate | 30,547 | 26,349 (87%) | 3,807 (48%) | 7,419 (100%) | 7,425 (100%) | 7,698 (100%) | <0.001 |
| Phenoage-Age advance accelerate | 30,547 | 14,662 (46%) | 3,524 (43%) | 3,137 (39%) | 3,935 (50%) | 4,066 (51%) | <0.001 |
| OA-group | 30,547 |  |  |  |  |  | <0.001 |
| Non-Osteoarthritis |  | 26,625 (86%) | 7,359 (91%) | 6,704 (89%) | 6,605 (87%) | 5,957 (75%) |  |
| Osteoarthritis |  | 3,922 (14%) | 646 (8.7%) | 715 (11%) | 820 (13%) | 1,741 (25%) |  |
| ^1^N not Missing (unweighted) | | | | | | | |
| ^2^median (IQR) for continuous; n (%) for categorical | | | | | | | |
| ^3^Wilcoxon rank-sum test for complex survey samples; chi-squared test with Rao & Scott's second-order correction | | | | | | | |

Table S2 pheno-Age advance quantile

| Characteristic | N^1^ | Overall  N = 30547 (100%)^2^ | Q1  N = 7494 (25%)^2^ | Q2  N = 7163 (25%)^2^ | Q3  N = 7362 (25%)^2^ | Q4  N = 8528 (25%)^2^ | P Value^3^ |
| --- | --- | --- | --- | --- | --- | --- | --- |
| Age (Years) | 30,547 | 45.72 ± 16.69 | 45.82 ± 15.93 | 44.57 ± 16.43 | 44.82 ± 16.64 | 47.66 ± 17.54 | <0.001 |
| Age-group (Years) | 30,547 |  |  |  |  |  | <0.001 |
| 20-44 |  | 14,294 (49%) | 3,497 (48%) | 3,640 (51%) | 3,658 (52%) | 3,499 (45%) |  |
| 45-59 |  | 7,603 (28%) | 1,996 (31%) | 1,737 (28%) | 1,756 (27%) | 2,114 (27%) |  |
| ≥60 |  | 8,650 (23%) | 2,001 (21%) | 1,786 (20%) | 1,948 (21%) | 2,915 (28%) |  |
| Gender | 30,547 |  |  |  |  |  | <0.001 |
| Male |  | 15,637 (51%) | 2,765 (37%) | 3,709 (52%) | 4,319 (58%) | 4,844 (56%) |  |
| Female |  | 14,910 (49%) | 4,729 (63%) | 3,454 (48%) | 3,043 (42%) | 3,684 (44%) |  |
| Race-group | 30,547 |  |  |  |  |  | <0.001 |
| Non-Hispanic White |  | 13,495 (68%) | 3,243 (69%) | 3,325 (71%) | 3,317 (68%) | 3,610 (64%) |  |
| Non-Hispanic Black |  | 5,663 (9.9%) | 1,055 (7.4%) | 1,104 (7.8%) | 1,413 (10%) | 2,091 (14%) |  |
| Mexican American |  | 5,023 (9.3%) | 1,371 (8.9%) | 1,175 (8.7%) | 1,214 (10%) | 1,263 (9.5%) |  |
| Other/multiracial |  | 3,443 (7.2%) | 1,051 (9.1%) | 867 (7.0%) | 768 (6.4%) | 757 (6.3%) |  |
| Other Hispanic |  | 2,923 (5.6%) | 774 (5.4%) | 692 (5.5%) | 650 (5.2%) | 807 (6.1%) |  |
| Education-group | 30,547 |  |  |  |  |  | <0.001 |
| Above High School |  | 17,161 (64%) | 4,474 (68%) | 4,186 (66%) | 4,184 (64%) | 4,317 (57%) |  |
| Below High School |  | 6,526 (14%) | 1,534 (12%) | 1,395 (12%) | 1,542 (14%) | 2,055 (17%) |  |
| High School |  | 6,860 (22%) | 1,486 (19%) | 1,582 (21%) | 1,636 (22%) | 2,156 (27%) |  |
| Smoke-group | 30,547 |  |  |  |  |  | <0.001 |
| Current smoker |  | 6,113 (19%) | 791 (11%) | 1,206 (16%) | 1,677 (21%) | 2,439 (29%) |  |
| Former smoker |  | 7,005 (24%) | 1,655 (24%) | 1,695 (24%) | 1,606 (22%) | 2,049 (24%) |  |
| Never smoker |  | 17,429 (57%) | 5,048 (65%) | 4,262 (60%) | 4,079 (56%) | 4,040 (47%) |  |
| Drink-group | 30,547 |  |  |  |  |  | <0.001 |
| <12 drinks/year |  | 27,391 (91%) | 6,917 (94%) | 6,555 (93%) | 6,568 (91%) | 7,351 (88%) |  |
| ≥12 drinks/year |  | 3,156 (8.7%) | 577 (6.4%) | 608 (7.4%) | 794 (9.2%) | 1,177 (12%) |  |
| BMI (kg/m^2^) | 30,547 | 28.78 ± 6.48 | 26.31 ± 4.79 | 27.90 ± 5.66 | 29.15 ± 6.15 | 31.74 ± 7.69 | <0.001 |
| BMI-group(kg/m^2^) | 30,547 |  |  |  |  |  | <0.001 |
| Normal |  | 9,106 (31%) | 3,121 (44%) | 2,362 (34%) | 1,954 (26%) | 1,669 (18%) |  |
| Obesiy |  | 21,441 (69%) | 4,373 (56%) | 4,801 (66%) | 5,408 (74%) | 6,859 (82%) |  |
| WC (cm) | 30,547 | 98.63 ± 15.99 | 91.77 ± 12.53 | 96.52 ± 14.29 | 99.81 ± 15.21 | 106.41 ± 17.79 | <0.001 |
| WC-group (cm) | 30,547 |  |  |  |  |  | <0.001 |
| Exceeds Standard |  | 16,592 (54%) | 3,278 (42%) | 3,552 (49%) | 4,058 (57%) | 5,704 (69%) |  |
| Normal |  | 13,955 (46%) | 4,216 (58%) | 3,611 (51%) | 3,304 (43%) | 2,824 (31%) |  |
| SBP (mmHg) | 30,547 | 121.11 ± 16.36 | 118.57 ± 15.85 | 120.30 ± 15.86 | 121.03 ± 15.88 | 124.55 ± 17.24 | <0.001 |
| Total Cholesterol (mg/dL) | 30,547 | 192.00 ± 41.38 | 197.18 ± 38.81 | 191.62 ± 39.42 | 191.31 ± 40.67 | 187.89 ± 45.72 | <0.001 |
| Glycated hemoglobin (%) | 30,547 | 5.56 ± 0.89 | 5.33 ± 0.46 | 5.38 ± 0.48 | 5.49 ± 0.61 | 6.02 ± 1.43 | <0.001 |
| Albumin (g/dL) | 30,547 | 4.29 ± 0.32 | 4.39 ± 0.28 | 4.33 ± 0.29 | 4.29 ± 0.31 | 4.15 ± 0.35 | <0.001 |
| Creatinine (mg/dL) | 30,547 | 2.88 ± 0.24 | 2.78 ± 0.15 | 2.86 ± 0.16 | 2.91 ± 0.18 | 2.98 ± 0.35 | <0.001 |
| Alkaline phosphatase (U/L) | 30,547 | 66.02 ± 22.02 | 60.01 ± 16.89 | 63.13 ± 17.61 | 66.34 ± 20.74 | 74.59 ± 28.22 | <0.001 |
| Blood urea nitrogen (mg/dL) | 30,547 | 13.33 ± 4.94 | 12.67 ± 4.00 | 13.08 ± 3.96 | 13.39 ± 4.59 | 14.19 ± 6.60 | <0.001 |
| Serum glucose (mg/dL) | 30,547 | 5.51 ± 1.85 | 4.95 ± 0.68 | 5.16 ± 0.82 | 5.35 ± 1.04 | 6.58 ± 3.15 | <0.001 |
| C-reactive protein (mg/dL) | 30,547 | 1.15 ± 0.11 | 1.14 ± 0.07 | 1.14 ± 0.08 | 1.15 ± 0.10 | 1.17 ± 0.16 | <0.001 |
| Lymphocyte (%) | 30,547 | 30.32 ± 8.00 | 34.17 ± 7.39 | 31.38 ± 7.23 | 29.39 ± 7.25 | 26.33 ± 8.00 | <0.001 |
| Mean cell volume (fL) | 30,547 | 89.49 ± 5.33 | 89.47 ± 4.35 | 89.76 ± 4.71 | 89.75 ± 4.95 | 88.98 ± 6.92 | 0.003 |
| White blood cell count  (1000 cells/uL) | 30,547 | 7.28 ± 2.22 | 6.04 ± 1.43 | 6.81 ± 1.53 | 7.53 ± 1.78 | 8.74 ± 2.89 | <0.001 |
| Red cell distribution width (%) | 30,547 | 13.27 ± 1.19 | 12.56 ± 0.64 | 12.97 ± 0.66 | 13.32 ± 0.74 | 14.23 ± 1.64 | <0.001 |
| Hypertension-group | 30,547 |  |  |  |  |  | <0.001 |
| Hypertension |  | 9,612 (29%) | 1,759 (22%) | 1,929 (25%) | 2,195 (28%) | 3,729 (41%) |  |
| Non-Hypertension |  | 20,935 (71%) | 5,735 (78%) | 5,234 (75%) | 5,167 (72%) | 4,799 (59%) |  |
| DM-group | 30,547 |  |  |  |  |  | <0.001 |
| DM |  | 3,299 (8.3%) | 329 (2.9%) | 397 (4.5%) | 596 (6.2%) | 1,977 (20%) |  |
| Non-DM |  | 27,248 (92%) | 7,165 (97%) | 6,766 (96%) | 6,766 (94%) | 6,551 (80%) |  |
| KDM-Age | 30,547 | 51 ± 19 | 51 ± 19 | 49 ± 18 | 50 ± 18 | 53 ± 20 | <0.001 |
| Phenoage-Age | 30,547 | 5.2 ± 5.1 | 5.1 ± 5.0 | 4.7 ± 4.6 | 5.1 ± 4.7 | 5.7 ± 5.8 | <0.001 |
| Phenoage-Age advance | 30,547 | 46 ± 18 | 41 ± 16 | 43 ± 16 | 46 ± 17 | 55 ± 19 | <0.001 |
| KDM-Age advance accelerate | 30,547 | 0.3 ± 5.2 | -5.1 ± 1.6 | -1.7 ± 0.7 | 1.0 ± 0.9 | 7.0 ± 5.3 | <0.001 |
| Phenoage-Age advance accelerate | 30,547 | 26,349 (87%) | 6,445 (87%) | 6,106 (86%) | 6,455 (88%) | 7,343 (86%) | 0.11 |
| OA-group | 30,547 | 14,662 (46%) | 0 (0%) | 0 (0%) | 6,134 (83%) | 8,528 (100%) | <0.001 |
| Non-Osteoarthritis | 30,547 |  |  |  |  |  | 0.013 |
| Osteoarthritis |  | 26,625 (86%) | 6,610 (87%) | 6,364 (87%) | 6,442 (86%) | 7,209 (83%) |  |
| DM-group |  | 3,922 (14%) | 884 (13%) | 799 (13%) | 920 (14%) | 1,319 (17%) |  |
| ^1^N not Missing (unweighted) | | | | | | | |
| ^2^median (IQR) for continuous; n (%) for categorical | | | | | | | |
| ^3^Wilcoxon rank-sum test for complex survey samples; chi-squared test with Rao & Scott's second-order correction | | | | | | | |
